# An Integrated Multi-Omics Analysis Defines Key Pathway Alterations in a Diet-Induced Obesity Mouse Model

**DOI:** 10.3390/metabo10030080

**Published:** 2020-02-25

**Authors:** Ulrik K. Sundekilde, Christian C. Yde, Anders H. Honore, Jessica M. Caverly Rae, Frank R. Burns, Pushkor Mukerji, Michael P. Mawn, Lotta Stenman, Yvonne Dragan, Kyle Glover, Henrik M. Jensen

**Affiliations:** 1 Department of Food Science, Aarhus University, Agro Food Park 48, DK-8200 Aarhus N, Denmark; 2 DuPont Nutrition Biosciences ApS, DK-8220 Brabrand, Aarhus, Denmark; 3 E. I. duPont de Nemours and Company, Inc., Haskell R&D Center, Newark, DE 19711, USA; 4 Global Health and Nutrition Science, DuPont Nutrition and Health, FI-02460 Kantvik, Finland

**Keywords:** obesity, multi-omics, metabolomics, transcriptomics, metagenomics, pathway analysis, systems biology

## Abstract

Obesity is a multifactorial disease with many complications and related diseases and has become a global epidemic. To thoroughly understand the impact of obesity on whole organism homeostasis, it is helpful to utilize a systems biological approach combining gene expression and metabolomics across tissues and biofluids together with metagenomics of gut microbial diversity. Here, we present a multi-omics study on liver, muscle, adipose tissue, urine, plasma, and feces on mice fed a high-fat diet (HFD). Gene expression analyses showed alterations in genes related to lipid and energy metabolism and inflammation in liver and adipose tissue. The integration of metabolomics data across tissues and biofluids identified major differences in liver TCA cycle, where malate, succinate and oxaloacetate were found to be increased in HFD mice. This finding was supported by gene expression analysis of TCA-related enzymes in liver, where expression of malate dehydrogenase was found to be decreased. Investigations of the microbiome showed enrichment of Lachnospiraceae, Ruminococcaceae, Streptococcaceae and Lactobacillaceae in the HFD group. Our findings help elucidate how the whole organism metabolome and transcriptome are integrated and regulated during obesity.

## 1. Introduction

Obesity is a global epidemic: approximately 39% of the world’s population was overweight or obese in 2016 [1]. Obese individuals are predisposed to the development of a number of chronic health disorders due to risk factors associated with obesity such as high blood glucose, excess body fat (predominantly abdominal), high blood triglycerides, abnormal cholesterol metabolism and increased blood pressure [2]. One of these chronic health disorders is metabolic syndrome, a cluster of conditions which is defined by abnormalities in energy utilization and storage, putting affected individuals at an increased risk of cardiovascular disease and type 2 diabetes. In addition, many consequences of poor lifestyle, such as chronic low-grade inflammation, intestinal permeability to bacterial endotoxins and changes in the intestinal microbiota have been proposed to be important contributors to these chronic health disorders [3,4,5]. In mice, a high fat diet has been shown to increase intestinal permeability [6,7], which is thought to be one of the mechanisms leading to low-grade inflammation.

The C57Bl/6J mouse is a commonly used model for the study of obesity and its consequences. These mice will develop glucose resistance by several weeks of age, followed by insulin intolerance and Type II diabetes [8]. The progression of this phenotype is exacerbated by the feeding of a high-fat diet, thus this mouse strain is a commonly used model for diet-induced obesity. Characteristic changes in blood parameters and microscopic and macroscopic tissue and organ findings have been described [9,10]. However, a systems biology perspective to these observations has not been applied.

Due to the multifactorial and complex nature of metabolic disorders, novel techniques should be employed to decipher global effects on interactions between organs, metabolites, and microbiota. Systems biology is the mathematical modelling of complex biological systems. The different molecular levels in an organism can be divided systematically into genomics, transcriptomics, proteomics, metabolomics and metagenomics. Genomics is the evaluation of an individual’s complete genome, including the mapping and functional analysis of individual genes, whereas transcriptomics is the measurement of the expression of these genes at any given time. Translation of these genes lead to protein expression as measured by proteomics. Proteins convert biological active compounds (metabolites) into other metabolites. Thus, metabolomics is the evaluation of metabolites in a biological matrix made complicated by the fact that metabolites are in a dynamic equilibrium and respond to external and internal factors, e.g., nutrients, stress, drugs, genes, microbiota. Finally, metagenomics is the characterization of gut microbial communities. Combining all these cross-sectional analyses in the same animals enables the analysis of interactions between multiple metabolite networks and gene expression markers across multiple tissues or locations and possible effects of microbial members on biological homeostasis.

In the current study, combinations of RNA transcriptomics in tissues, metabolomics of biofluids and tissues and metagenomics analyses were investigated in a high-fat diet (HFD)-fed mouse model (C57BL/6J). Overall, the aim was to elucidate the underlying molecular links between a HFD and the development of obesity.

## 2. Results

### 2.1. In-Life Parameters and Organ Weights

**Figure 1:**
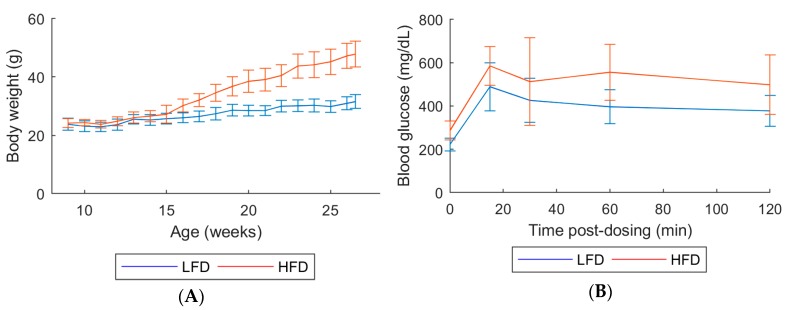
(**A**) Body weight measured weekly. (**B**) Glucose tolerance test at age 18 weeks. Animals in each group: 10 males. Error bars indicate the standard deviation of biological replicates (*n* = 10). Abbreviations: LFD, low-fat diet; HFD, High-fat diet.

All mice survived until their scheduled sacrifice. Over the course of 20 weeks of treatment, HFD mice exhibited higher body weights and reduced glucose tolerance as seen by the elevated glucose levels, compared with low-fat diet (LFD) mice (Figure 1). 

Mean terminal body weights in HFD mice were approximately 1.57-fold higher than in LFD mice (Figure 1A and Table S1). Absolute weights for all organs assessed were increased relative to LFD (1.20-fold higher for kidney, 1.4-fold higher for liver and 1.3-fold higher for spleen; Table S1). 

### 2.2. Gene Expression

**Figure 2:**
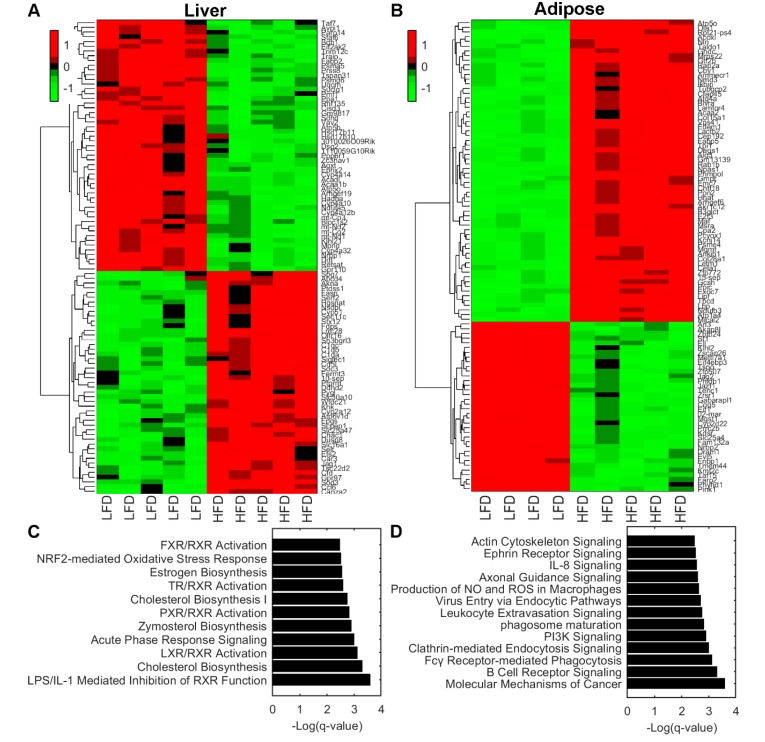
(**A**,**B**) Hierarchical clustering of top 100 significant differentially expressed genes in liver tissue (**A**) and adipose tissue (**B**). (**C**,**D**) Pathway enrichment analysis using Ingenuity Pathways Analysis software of liver tissue (**C**) and adipose tissue (**D**). Statistical significance is expressed as *q* values of a right-tailed Fisher’s Exact test with multiple hypothesis correction based on the Benjamini–Hochberg approach. Abbreviations: LFD, low-fat diet; HFD, High-fat diet.

Gene expression analyses on liver and adipose tissue showed alterations due to LFD and HFD feeding (Figure 2). In liver tissue, there were 861 differentially expressed genes of which 501 where upregulated and 360 downregulated. In adipose tissue, a total of 3376 genes were differentially expressed where 2355 were upregulated and 1021 downregulated. The top 100 significant differentially expressed genes were plotted in a heat map analyzed by hierarchical clustering in liver (Figure 2A) and adipose tissue (Figure 2B). 

To gain mechanistical insight into which biological pathways are enriched, we performed pathway enrichment analysis on liver. The analysis showed that biochemical pathways LPS/IL-1 mediated inhibition of RXR, Cholesterol biosynthesis, LXR/RXR activation, Acute phase response signaling, and Zymosterol biosynthesis were enriched (Figure 2C). These pathways are involved in the regulation of lipid metabolism, inflammation, and cholesterol metabolism. Adipose tissue exhibited significantly altered pathways involved in cell cycle, inflammation, and energy metabolism. These include molecular mechanisms of cancer, B cell receptor signaling, Fcγ Receptor-mediated phagocytosis, Clathrin-mediated endocytosis signaling, and PI3K signaling pathways (Figure 2D).

Further gene expression studies on liver tissue revealed that Fh1, a gene encoding the enzyme responsible for conversion of fumarate into malate, was significantly increased (*q* = 0.04), while the expression of Mdh2, which converts malate into oxaloacetate, was decreased (*q* = 0.037; Table S2). Likewise, Mdh1 showed a tendency towards decreased expression (Table S2); however, this difference did not reach significance.

### 2.3. Metabolomics

**Figure 3:**
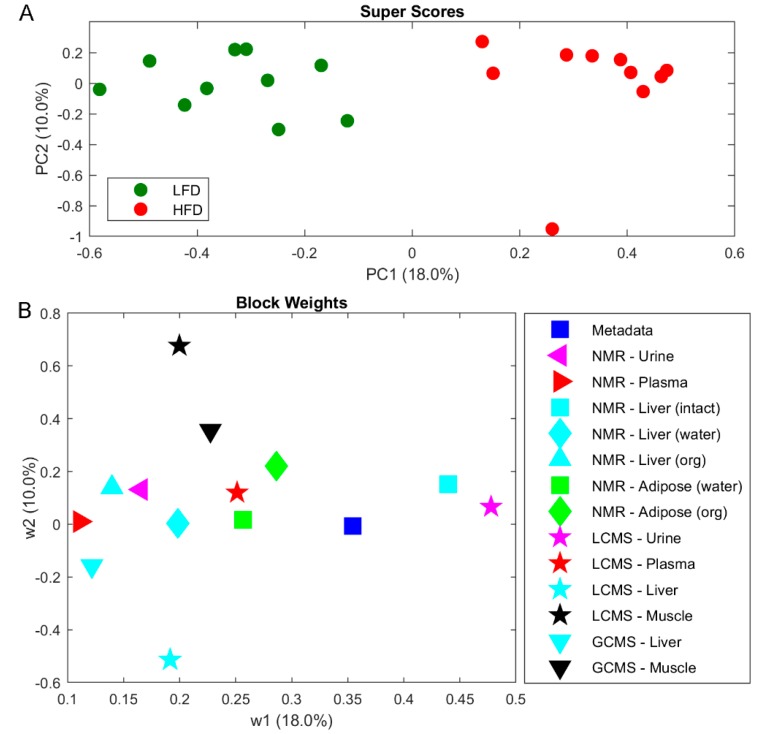
Multi-block principal component analysis (PCA) on metabolomics data from biofluids and tissues as indicated. (**A**) Super scores scatter plot. (**B**) Block weights of biofluids and tissues. Abbreviations: LFD, low-fat diet; HFD, High-fat diet.

Metabolite profiling was performed on tissues and biofluids by multiple analytical techniques to evaluate the whole metabolome of C57BL/6J mice. A combined analysis of urine, plasma, liver, adipose tissue, and muscle tissues were completed by NMR, LC-MS and GC-MS. In an effort to combine the metabolomes across tissue and biofluids a multi-block PCA (MBPCA) approach was undertaken (Figure 3). Mice fed LFD and HFD were perfectly separated by the MBPCA method (Figure 3A). The block weights show the importance of the loading within each block on the overall grouping (Figure 3B), with urine and liver shown to contain most differential data supporting the LFD/HFD differences. 

**Figure 4:**
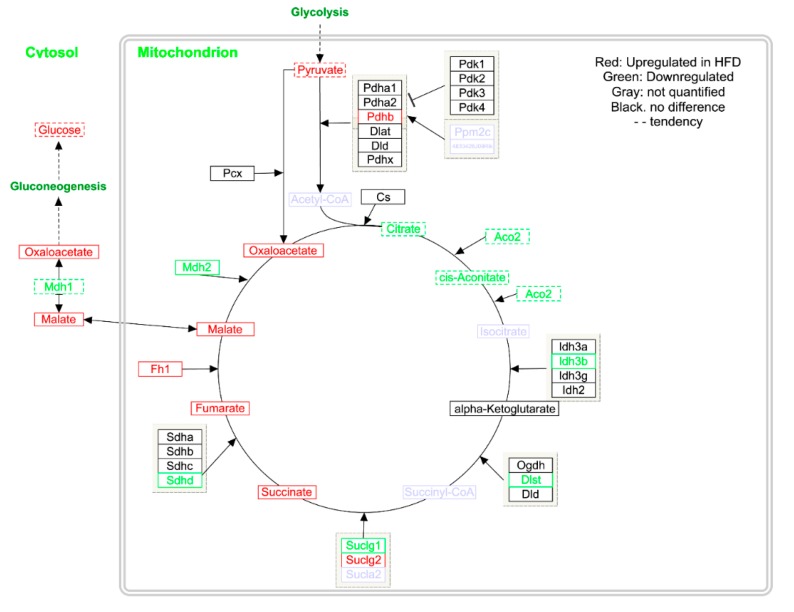
The tricarboxylic acid (TCA) cycle showing differences in gene expression and metabolite abundances in mouse liver. Upregulated in HFD are shown in red, and those that were downregulated are shown in green, genes and metabolites not quantified are shown in gray. Data is shown in Table 1 and Table S2.

Overall, most of the individual block scores showed a separation between LFD and HFD (Figure S1). The most influential block is the LCMS urine (Figure 3), where also a clear separation of LFD and HFD is observed (Figure S1Q,R). The next most influential block is intact liver tissue by HRMAS spectroscopy, where differences mainly can be attributed resonances from increased lipid accumulation in liver from HFD mice (Figure S1G,H). Increased amounts of lipid accumulation in HFD mice is also evident in adipose tissue extracted in CHCl_3_ (Figure S2O,P). In aqueous extracted liver samples succinate and malate was found to be significantly elevated in HFD-fed mice (respectively *q* = 0.04 and *q* = 0.008; Table 1 and Figure S1I,J,Y,Z). Consequently, biological pathways involving succinate and malate were examined in more detail. Both are key metabolites in the tricarboxylic acid (TCA) cycle and several intermediate steps on metabolite and gene expression levels in the mitochondrial TCA cycle were found to be significantly altered (Figure 4; Table 1). Metabolite profiling revealed that HFD-mice livers exhibited increased abundances of fumarate (*q* < 0.01), oxaloacetate (*q* = 0.035), whereas citrate and cis-aconitate levels showed a tendency to be decreased (Table 1; Figure 4). Urinary TCA metabolites are indicated to be elevated in HFD mice (Figure S1D).

### 2.4. Metagenomics

16S rDNA sequencing of fecal samples collected at six time points from week 1 to week 26 was performed and clustered into operational taxonomic units (OTUs) to investigate the effects of microbiota composition and LFD/HFD treatment. 

**Figure 5:**
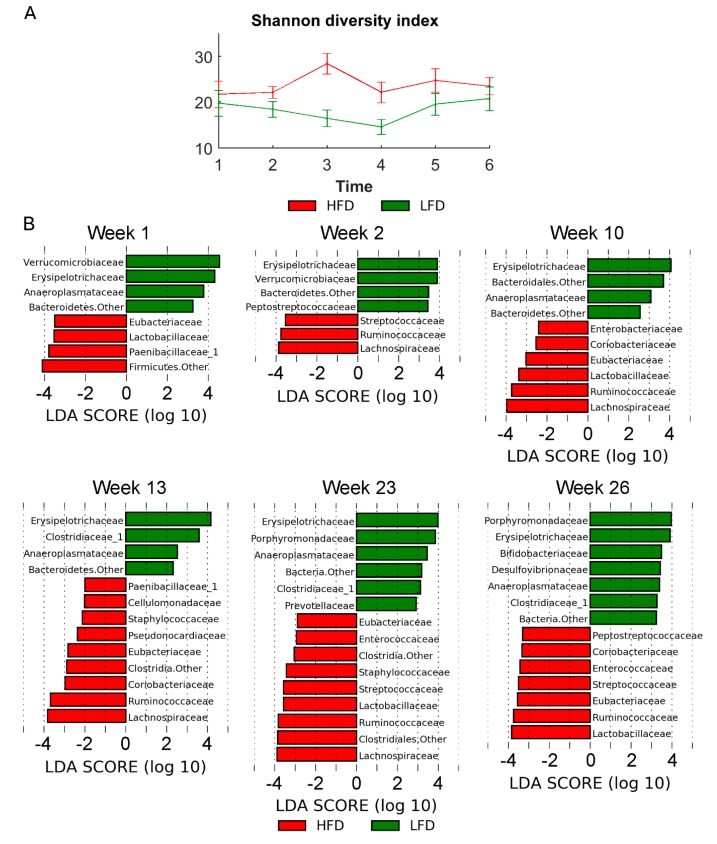
(**A**) α-diversity (Shannon diversity index). (**B**) Histograms showing the log-transformed Linear discriminant analysis (LDA) scores computed with Linear discriminant analysis effect size (LEfSe) for significantly different bacterial taxa between diet groups at the different sampling time points. A positive LDA score indicates enrichment in LFD, whereas a negative LDA score indicates enrichment in HFD. The LDA score indicates the effect size and ranking of each bacterial taxon. Statistical significance was evaluated using the Kruskal–Wallis test (alpha < 0.05) and a log-transformed LDA score with a threshold of 2.0. Error bars indicate the standard deviation of biological replicates (*n* = 10). Abbreviations: LFD, low-fat diet; HFD, High-fat diet.

The metagenomics analysis showed similar richness estimated by the Shannon diversity index (number of species in the community) across the two diets (Figure 5A). In order to investigate differential microbiota at different time points and between diets, the linear discriminant analysis (LDA) effect size (LEfSe) method was used. 

**Figure 6:**
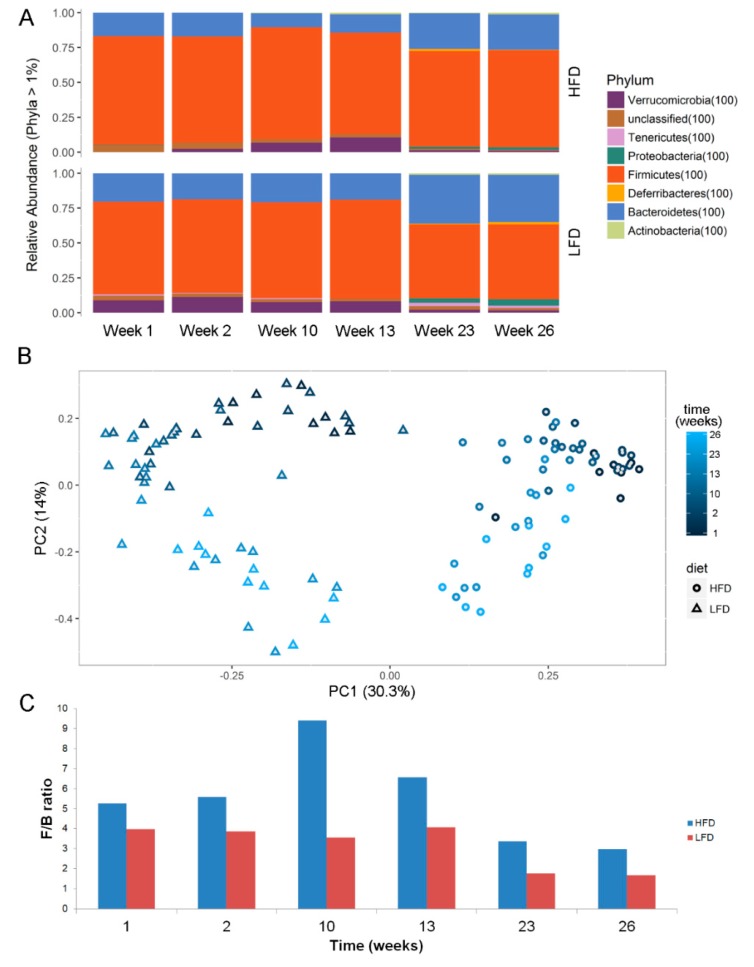
(**A**) Phyla abundance and (**B**) Unsupervised ordination using Bray–Curtis dissimilarity-based principal coordinates plot showing clusters of diets (PC1) and time (PC2). (**C**) ratios of relative abundance of firmicutes and bacteriodetes (F/B ratio) at different time-points. Abbreviations: LFD, low-fat diet; HFD, High-fat diet.

In early time points (week 1–2), less bacterial taxa (OTUs) were found to be differentially enriched. At week 23 with the most diverse distribution, anaeroplasmataceae, clostridiaceae_1, erysipelotrichaceae, porphyromonadaceae, and prevotellaceae were found to be enriched in LFD, whereas enterococcaceae, eubacteriaceae, lachnospiraceae, lactobacillaceae, ruminococcaceae, staphylococcaceae, and streptococcaceae were found to be enriched in HFD mice (Figure 5B). Examining the microbiota at phyla level also showed significant differences between microbiota in LFD/HFD mice (Figure 6A). HFD mice had an increased ratio of Firmicutes/Bacteriodetes at all time points (Figure 6C). Consumption of HFD consistently modified the gut microbiota as the two diets formed two clusters by unsupervised ordination using Bray–Curtis dissimilarity-based principal coordinates analysis (PCoA; PC1; Figure 6B). In addition, time dependent clusters can be observed within each diet-cluster showing a consistent effect of time (PC2; Figure 6B). These shifts are also visible at the phylum level (Figure 6A).

## 3. Discussion

Obesity, as a major risk factor for metabolic syndrome as well as other human diseases, involves alterations in numerous biological pathways, including gene and protein regulation, which in turn affects the production of metabolites. Moreover, predisposition to the development of obesity is complicated and affected by genetics, lifestyle, diet and other factors. Different profiles of gut microbiota have been associated with lean and fat body types, making the gut microbiome a possible contributing factor in obesity [11,12]. Due to the complicated causes and effects of obesity, a combined examination of the different biological levels within an organism through a systems biology analysis is advantageous. To this end, animal models are a powerful resource in the cross-sectional study of complex syndromes using today’s omics-approaches [9]. The use of inbred mouse strains minimizes confounding effects of genetic differences thereby reducing the multifactorial nature of the syndrome to a factor less. Moreover, mouse models enable discovery of effects on metabolites, gene expression, genetics, and microbiota from tissues that cannot be sampled from healthy human volunteers [9,13,14,15]. Numerous models of diet-induced obesity and diabetes have been developed. The HFD C57Bl/6 mouse model is one of the most commonly studied animal models of diet-induced obesity. Accordingly, this animal model is useful for the elucidation of mechanisms due to HFD-induced obesity on a systems biological level. 

In the present study, we investigated the effect of HFD on C57Bl/6 mice by acquiring gene expression and metabolomics data in multiple tissues as well as metagenomics data on gut microbiota. By integrating all cross-sectional data, we found several pathways at different, but derivative, biological levels altered due to diet-induced obesity in the model. Clinical data verified that the phenotype of the HFD mice used in this study successfully modeled diet induced obesity. Namely, body and organ weights in the HFD mice were greater than in the LFD mice, and glucose tolerance was reduced in HFD mice compared to LFD mice. 

Gene expression analysis revealed that adipose tissue had the most differentially expressed genes, many related to inflammation. As adipose tissue is a collection of adipocytes, stromal cells, tissue macrophages and migrating inflammatory cells, part of this response is likely attributable to the presence of differential populations of inflammatory cells in the adipose tissues of the HFD mice rather than a change in gene expression in adipocytes. Similarly, genes related to inflammation were also identified by enrichment analysis in the liver. Inflammation associated with obesity is believed to play a role in the development of comorbidities such as metabolic syndrome, and increased inflammatory cytokines have been shown to be related to insulin resistance and type 2 diabetes [16]. Thus, increases in the expression of inflammation-related genes in the adipose tissue and liver of HFD mice is consistent with the clinical presentation of these mice and models the postulated human condition. 

Multi-block PCA is a tool to explore structural differences and similarities in multi-block data, as the case of metabolomics data across tissues and biofluids [17]. In order to comprehensively characterize the whole body metabolic profile, multiple analytical techniques and extraction solvents were used to generate urine, plasma, liver, adipose, and muscle metabolomes. Each analytical run was treated as a block of data in MB-PCA. A major difference between LFD and HFD mice was found in levels of liver TCA cycle intermediates. Here, we found fumarate, malate, and oxaloacetate to be increased in the liver of HFD mice, and gene expression of enzymes related to the TCA cycle were found to be upregulated or downregulated corroborating the identified differences in metabolite levels. The observed fold-changes in gene-expression was small, yet the biological relevance and interpretation remains intact, as there is a direct relationship between metabolites and enzymes. The TCA pathway in the liver of DIO mice has previously been studied and observed to exhibit overall lower abundance of TCA intermediates [18]. In our study, citrate and cis-aconitate along with gene expression of Aco2 were upregulated in LFD mice in agreement with the previous literature. However, we observed that abundance of succinate, fumarate, malate and oxaloacetate were increased in HFD mice, which indicate impaired hepatic TCA function. The mitochondrial TCA cycle has previously been examined in insulin resistant mice [19], where results showed an increased TCA function similar to the results we obtained in the present study. Urinary succinate has been shown to be increased in HFD mice suggesting overall upregulation of the TCA pathway [20]. The increased levels of urinary TCA metabolites observed in the present study support these findings. The TCA cycle is a key metabolic pathway that connects carbohydrate, protein and fat metabolism by the oxidation of pyruvate into energy and CO_2_. These findings indicate elevated gluconeogenesis consistent with the pyruvate-driven gluconeogenesis and increased TCA cycle flux that have been observed as a consequence of diet-induced obesity independent of diet [21]. 

A multi-omics study by Kieffer et al. investigated the effect of high-fat diet supplemented with resistant starch, a form of starch that passes through the small intestine unabsorbed and is mostly degraded by gut microbes [22]. Liver TCA metabolites fumarate and malate levels were found to be significantly decreased when mice were fed diets supplemented with resistant starch [22]. This suggests that the increased levels of liver TCA metabolites found in the present study could be related to changes in the gut microbiota. In general, multi-omics studies have shown a great potential to investigate the interplay between diet, organism and microbial community.

Alterations in gut microbiota due to obesity are known to increase the Firmicutes/Bacteriodetes ratio as first shown by Gordon’s lab [23]. In this study we observe a higher F/B ratio in the HFD group compared to LFD at all time points, with the biggest difference at week 10. Recently, the Turnbaugh team performed a meta-analysis of 25 deposited murine HFD studies using machine learning to predict HFD intake in mice from 16S rRNA Sequencing data [24]. After removal of Lactococcus, which seem to be a contaminant from the diet, the most informative Operational Taxonomic Units (OTUs) predicting HFD diets in mice studies were three clades of Lachnospiraceae, Ruminococcaceae UCG-014, and S24-7 Muribaculaceae. According to this approach, these three OTUs outperformed the F/B ratio in predicting the HFD diets. In accordance, Lachnospiraceae and Ruminococcaceae are among the OTUs enriched in our study. A large US human cohort study reported the abundance of OTUs Streptococcaceae and Lactobacillaceae to be increased with obesity, while OTUs within Clostridia were decreased [25]. In addition to Lachnospiraceae and Ruminococcaceae, we observed enrichment of Streptococcaceae and Lactobacillaceae in the murine HFD group. Interestingly, Bisanz et al. also indicated that the prediction from OTU taxonomic signatures seem to be translatable between humanized mice and humans. However, it remains unclear if the association of the F/B ratio observed in animal studies can be translated into humans [26].

In conclusion, our findings elaborate on the regulation between different biological levels in systems biology. Notably, TCA cycle was found to be altered in HFD-mice as multiple metabolites and genes were found to be dysregulated including co-elevated levels of malate and fumarate. This increase in TCA metabolites was coordinated with a decreased expression of malate dehydrogenase, responsible for converting malate into oxaloacetate. However, associating the observed host metabolic changes to changes in the microbiome remains a challenge. But the use of animal models to elucidate the biological response of the entire organism is essential to identify molecular mechanisms of diet-induced obesity.

## 4. Materials and Methods 

### 4.1. Animals

The colony of C57Bl/6J mice was maintained at The Jackson Laboratory (JAX; Bar Harbor, ME, USA). At 6 weeks of age, 20 males were divided into 2 groups by the breeding facility, and fed purified diets containing either 60% fat (D12492, high fat diet; HFD) or 10% fat (D12450B, low fat diet; LFD), manufactured by Research Diets, Inc (New Brunswick, NJ, USA). At 8 weeks of age, 10 males from each group were shipped to DuPont Haskell Global Center for Health Sciences (Newark, DE, USA), an AAALAC-accredited test facility, and continued to be fed (ad libitum) the respective HFD or LFD diet provided by the breeder. Animals were maintained in accordance with the Guide for the Care and Use of Laboratory Animals (National Research Council, 2011), and the protocol was approved by the DuPont Haskell Institutional Animal Care and Use Committee (IACUC), protocol number AT311-P. All animals were housed individually in solid bottom caging with bedding and nesting material as environmental enrichment, and tap water was provided ad libitum. Animal rooms were maintained on a 12-h light/dark cycle (fluorescent light), at 22 ± 4 °C and a relative humidity of 50% ± 20%. Mice were sacrificed at 26 weeks of age, after 20 weeks on their respective diets. 

Body weights were measured weekly. A glucose tolerance test (GTT) was performed on all animals at 9, 11, 14, 15, 18, and 20 weeks of age. Each animal was fasted for 6 h and then administered 50 mg glucose by oral gavage (0.125 mL of a 40% solution). Glucometer readings were recorded prior to dosing and at approximately 15, 30, 60, and 120 min post-dosing. Blood (≥ 0.3 µL) was obtained by pricking the tail vein with a sterile needle. Glucose was measured using the AlphaTrak 2 glucometer (Zoetis; Parsippany, NJ, USA). 

Fecal samples were collected from the animal shipping crates upon arrival at DuPont Haskell laboratory, and then from each animal twice during the 1st two weeks at the facility. At 10, 13, and 23 weeks of age, animals were fasted in metabolism cages (with access to water) for at least 6 h prior to collection of blood, urine, and fecal samples. Blood was collected from the tail vein at weeks 10 and 13 and processed to plasma, and collected from the orbital sinus (under anesthesia via isoflurane inhalation) at week 23 and processed to plasma. At 26 weeks of age, animals were fasted for at least 15 h for collection of urine and fecal samples, and then euthanized by exsanguination following isoflurane inhalation. During exsanguination, blood was collected via vena cava and processed to plasma. Samples were frozen at < −60 °C until analyzed for metagenomics (feces) or metabolomics (plasma and urine).

Sections of liver, gastrocnemius muscle, and epididymal fat were flash frozen and stored at −80 °C until analyzed for metabolomics or transcriptomics. 

### 4.2. Gene Expression, Data Generation and Analysis

Each RNA isolation was performed from 30mg of tissue. RNA was liberated by tissue homogenization in the presence of Trizol on a Genogrinder. The homogenate was extracted once with phenol chloroform and the aqueous phase was further processed using RNAeasy columns with on column DNAase treatment (Qiagen). RNA seq libraries were generated using the Illumina TruSeq^®^ stranded mRNA Kit (Illumina, San Diego, CA, USA). Sequencing was performed by generating 50 base reads on an Illumina HiSeq 2500 instrument (Illumina). Reads were aligned to the Mus_musculus_GRCm38.p3 reference genome. Sequences were summed on the transcript level, normalized to relative parts per Kilobase per 10 Million (RPKtM) differential expression analysis performed in the using the GeneData package. Higher level integration of differential gene expression data was performed using Ingenuity Pathway Analysis program (IPA; Qiagen, Germantown, MD). Pathway enrichment analyses were performed with IPA using Fisher’s exact test with multiple hypothesis correction based on the Benjamini–Hochberg approach (*q* < 0.05). Hierarchical clustering of gene expression data was performed in MATLAB 2017b (Mathworks, Natick, MA, USA).

### 4.3. Metabolomics

#### 4.3.1. NMR Spectroscopy

The NMR measurements were performed on a 600 MHz Bruker Avance spectrometer (Bruker Biospin, Rheinstetten, Germany) operating at a frequency of 600.13 MHz for ^1^H nucleus. 

Plasma: the plasma samples were thawed and 25 µL plasma sample were mixed with 10 µL deuterium oxide. ^1^H NMR spectra were recorded at 310 K using a 1.7 mm TXI (triple resonance inverse) probe with the CPMG ‘PROJECT’ (Periodic Refocusing of J-Evolution by Coherence Transfer) sequence [27] and pre-saturation. A total of 128 scans collected into 32K data points were acquired with a spectral width of 17.35 ppm, a total spin echo delay of 160 ms (4nτ), a spin-echo delay of 0.2 ms (τ), a recycle delay of 2 s and an acquisition time of 1.57 s. An exponential line broadening function of 0.3 Hz was applied to the free induction decay prior to the Fourier transformation. Each spectrum was referenced to the anomeric signal of α-glucose at 5.23 ppm.

Urine: 400 µL of urine were mixed with 200 µL D_2_O containing 0.05% 3-(Trimethylsilyl)propionic-2,2,3,3-d4 acid sodium salt (TSP). A standard 1D Noesy experiment with pre-saturation (Bruker “noesygppr1d” sequence) was used to acquire ^1^H NMR spectra at 298 K. A total of 64 scans collected into 75K data points were acquired with a spectral width of 24.03 ppm, a recycle delay of 5 s and an acquisition time of 2.60 s. An exponential line broadening function of 0.8 Hz was applied to the free induction decay prior to the Fourier transformation.

Tissue extracts: A methanol/chloroform/water (1:1:1) extraction was performed, and the tissue extracts were placed at 4 °C overnight for separation. The tissue extracts were centrifuged (1400× *g*, 30 min, 4 °C), and the methanol-water and chloroform phases were separated for NMR, GCMS and LCMS analysis (ratio: 2:1:1) and desiccated in a vacuum centrifuge and stored at −80 °C. Prior to NMR analysis, the methanol–water fractions (‘water’ is used to label these samples) were prepared by dissolving the pellet with 550 µL D_2_O, 25 µL H_2_O and 25 µl D_2_O containing 0.05% TSP, and the chloroform fractions (‘org’ is used to label these samples) were dissolved in 575 µL CHCl_3_-d and 25 µL CHCl_3_-d containing 0.05% Tetramethylsilane (TMS). ‘Water’ extracts from adipose tissue and liver: ^1^H NMR spectra were obtained at 295 K using a 1D Noesy experiment (noesygppr1d, Bruker sequence). A total of 128 scans collected into 64 K data points were acquired with a spectral width of 14.00 ppm, a recycle delay of 5 s and an acquisition time of 3.90 s. An exponential line broadening function of 0.8 Hz was applied to the free induction decay prior to the Fourier transformation. ‘Org’ extracts from adipose tissue and liver: ^1^H NMR spectra were obtained at 298 K using a single pulse sequence with a 30° flip angle (zg30, Bruker sequence). A total of 64 scans collected into 64 K data points were acquired with a spectral width of 20.03 ppm, a recycle delay of 1 s and an acquisition time of 2.73 s

Intact liver (High Resolution Magic Angle Spinning (HR-MAS) analysis): A piece of each of the intact liver samples (still frozen) were packed at −20 °C in disposable pre-weighed 50 μL inserts (Bruker Biospin, Rheinstetten, Germany) followed by addition of 10 μL of D_2_O containing 0.05% TSP. Upon measurement, the insert (sample) was placed in a 4 mm zirconium rotor (Bruker BioSpin, Rheinstetten, Germany) and ^1^H NMR spectra were acquired with a CPMG experiment (cpmgpr1d, Bruker sequence) using a 4 mm HR-MAS probe (Bruker BioSpin, Rheinstetten, Germany). The acquisition parameters for the spectra were as follows: 5 kHz spin rate, 64 scans, a spectral width of 17.36 ppm with 32 K data points, a total spin−spin relaxation delay of 100 ms (2nτ), a spin−echo delay of 1 ms (τ), a recycle time of 3 s and an acquisition time of 1.57 s. An exponential line broadening function of 0.3 Hz was applied to the free induction decay prior to the Fourier transformation. Each spectrum was referenced to the anomeric signal of α-glucose at 5.23 ppm. Chenomx NMR Suite (Chenomx Inc., Edmonton, Alberta, Canada) was used to profile ^1^H spectra to extract metabolite identity and concentration. In addition, multivariate data analysis on ^1^H NMR data was also applied. Prior to multivariate analysis data was adjusted for minor chemical shifts using Icoshift [28]. For tissue samples data was normalized to sample weight. Uninformative spectral regions (residual water and spectral ends) were removed. Urine samples were normalized to 1-norm. Finally, the data was reduced by binning [29].

#### 4.3.2. LC-MS Analysis on Urine and Plasma

The LC-MS analysis of the urine and plasma sample matrices was performed using a Waters I-Class Acquity UPLC interfaced to a Thermo Q-Exactive high resolution accurate mass Instrument. Full Scan LC-MS spectra from 67–1000 Daltons at 35,000 resolution and data dependent MS^2^ spectra from 150 to 1000 Daltons at 17,500 resolution were collected for both positive and negative ion electrospray ionization (ESI) modes. Samples were analyzed using both reverse phase and HILIC chromatographic conditions. The reverse phase analysis was performed using a Waters Acquity BEH-C18 column with dimensions of 2.1 × 100 mm with 1.7 µm particle size. For the positive ion mode the UPLC mobile phase A was 0.1% formic acid in water and mobile phase B was 0.1% formic acid in 70:30 acetonitrile:methanol. For the negative ion mode the UPLC mobile phase A was 5 mM ammonium acetate in water and mobile phase B was 5 mM ammonium acetate in 70:30 acetonitrile:methanol. The UPLC binary pump flow rate was 0.4 mL/min with initial gradient of 2% B for 0.5 min then ramped up to 30%B at 7 min then ramped up to 100%B at 11 min and held until 13 min. At 13.1 min the %B was stepped changed to 2%B to re-equilibrate the column. The total run time was 15 min. The HILIC analysis was performed using a Merck SeQuant ZIC-cHILIC column with dimensions 2.1 × 150 mm with 3 µm particle size. For the positive and negative ESI modes the UPLC mobile phase A was 10mM ammonium acetate in 90:5:5 water:acetonitrile:methanol with 0.3% acetic acid, and mobile phase B was 10 mM ammonium acetate in 10:90 water:acetonitrile with 0.3% acetic acid. The UPLC binary pump flow rate was 0.4 mL/min with initial gradient of 99% B for 0.5 min then ramped down to 30%B at 20 min then ramped up to 99%B at 20.1 min to re-equilibrate the column. The total run time was 24 min.

The urine and plasma samples were thawed and 80 µL sample aliquots were added by pipette into 2-mL microcentrifuge tubes. Reagent blank samples containing 80 µL of 1:1 acetonitrile:methanol solvent were also prepared. Into each tube 320 µL of cold 1:1 acetonitrile:methanol protein precipitation extraction solvent was added by pipette and the samples were vortexed and centrifuged for 10 min at 4 °C. The supernatant was decanted into new 2-mL microcentrifuge tubes, and after breaking up the pellets the initial tubes were re-extracted with an additional 320 µL extraction solvent. The supernatant was combined with the first extract, and evaporated to dryness using a SpeedVac (Thermo Model SPD1010) at 45 °C. The residue was reconstituted with 160 µL of 1:1 methanol:water solvent, vortexed, centrifuged, and supernatant transferred to high recovery HPLC vials for analysis.

A pooled QC sample for each matrix was prepared by pipetting 30 µL of each of the final prepared samples from the low-fat and high-fat diet groups. Solvent blank QC samples were also prepared. The plasma and urine samples matrices were analyzed separately, and each batch included a randomized sequence, which included the low-fat, high-fat, reagent blank, and pooled QC samples with a total of 5 injections for each sample type. MS data were converted to mzXML files and uploaded to XCMS Online for data processing including peak detection using centWave algorithm, retention time correction, profile alignment, and isotope annotation [30]. Features were tentatively assigned by searching an internal database and HMDB [31] based on exact mass with a tolerance of 5 ppm.

#### 4.3.3. LC-MS Analysis on Tissue Samples

Methanol, Acetonitrile (LCMS grade) and formic acid, (LCMS grade) were purchased from Fisher Scientific (Hampton, NH, USA). All water employed was of freshly prepared Milli-Q quality (Merck Millipore, Billerica, MA, USA).

The methanol-water extracted pellets (see Section 3, Tissue Extracts) were reconstituted in pre-cooled (5 °C) 50 µL water/methanol v/v and mixed at 1400 ppm and 10 °C in an Eppendorf Thermomixer Comfort (Eppendorf Nordic ApS, Horsholm, Denmark). The dissolved samples were centrifuged at 12,000× *g* at 4 °C for 5 min. An aliquot of 40 µL supernatant is transferred to 300 µL injection vials. A pooled sample (MIX) was prepared by sampling and mixing 7 μL of each supernatant included in the study. 

The LC/MS system was equilibrated with a minimum of six replicate injections of the MIX sample prior to analyzing samples. Sample injections were performed in triplicate. Each set of replicates was placed in randomized blocks containing ten samples and one MIX sample. 

The LC/MS analysis was performed using an Agilent (Agilent Technologies, Waldbronn, Germany) modular 1290 ultra-performance liquid chromatography (UPLC) instrument coupled to a Bruker (Bruker Daltonics, Billerica, MA) maXis 4G single-quadrupole time-of-flight mass spectrometer (MS) via an electrospray interface. The UPLC was mounted with a Waters (Waters Corporation, Milford, MA, USA) HSS T3, 2.1 × 150 mm id column + 2.1 × 5 mm precolumn packed with 1.8-μm particles and maintained at 40 °C. Mobile phases were (A) water/formic acid 1000:1 *v/v* and (B) acetonitrile/formic acid 1000:1 *v/v*. Vials were kept at 5 °C in the autosampler prior to injection of 5 μL. Elution was performed with a flow of 450 μL/min and a gradient starting at 0% B at t = 0 and kept for 2 min, to 25% B at 6 min, to 80% at 10 min, to 90% B at 12 min and finally to 99% B and kept for 2 min; then back to 0% B over 0.1 min and maintained for 4.9 min. The electrospray interface with nebulizer at 2.5 bar and dry gas at 9.0 L/min at 200 °C was operated in both positive and negative mode (capillary voltage at 4200 V and 3500 V, respectively). Mass spectra in the range *m/z* 60–1650 were acquired with a frequency of 3 Hz. The *m/z* axis was calibrated with sodium formate clusters (solution of water/2-propanol/1 mol/L sodium hydroxide/formic acid 250:250:2.5:0.5 *v/v/v/v*) infused prior to each chromatographic run via a divert-valve loop setup. The instrument was controlled using Bruker Daltonics micrOTOFcontrol version 4.0 and acquired data was handled with Data Analysis version 4.3. 

Prior to feature extraction, chromatographic data of MIX samples (total ion chromatograms (TICs) and base peak chromatograms (BPCs)) were inspected visually for irregularities like drift in intensities and retention time. Bruker raw data files were converted into mzXML files by Bruker CompassXport v.3.0.9 (Bruker Daltonics). 

The mzXML files were imported to MzMine2, version 2.6 [32] for feature extraction. Peak detection was based on an *m/z* tolerance of 0.001 Dalton (Da) or 5 parts per million (ppm) and a peak duration time range of 0.025–0.35 min. Chromatograms were deconvoluted using the “local minimum search” algorithm, de-isotoped and peaks were aligned using the Join aligner algorithm. Peak lists were filtered using a criterion of a feature being detected in a minimum of three chromatograms. The peak list was gap filled and filtered for duplicate peaks with a retention time tolerance of 0.1 min and mass accuracy 0.001 Da or 5 ppm. The features, each representing one compound, at a given mz@RT, were tentatively assigned by searching an internal database and HMDB [31] based on exact mass with a tolerance of 5 ppm.

After processing, resulting feature tables were inspected by principal component analysis (mean centering, pareto) for grouping and dispersion of mix samples as well as plotting of first four principal components against run order for inspection of drift.

#### 4.3.4. GC-MS Analysis

Reagents and solvents for the GC/MS analysis were pyridine and N-methyl-N-trimethylsilyl-trifluoroacetamide (MSTFA) from Fisher Scientific (Hampton, NH, USA) as well as trimethylchlorosilane (TMCS) Sigma-Aldrich (St. Louis, MO, USA). Internal standards included sorbitol-13C6 Sigma-Aldrich (St. Louis, MO, USA) and in addition heptadecane and norvaline (Fisher Scientific, Hampton, NH, USA). Methoximation reagent was prepared by weigh-in of 0.5 g methoxyamine hydrochloride, Sigma-Aldrich (St. Louis, MO, USA), and 10 mL pyridine was added. Silylation reagent was prepared by adding 100 µL TMCS to 9.9 mL MSTFA. Methoximation was performed on approximately 1 mg of sample from the methanol-water extracted pellets (see Section 3, Tissue Extracts) as a batch process by addition of 20 µL methoximation reagent and reaction for 90 min at 37 °C, and 900 RPM on a Heidolph Vibramax 100 (Heidolph Instruments GmbH & Co. KG, Schwabach Germany). Subsequently, 1 µL internal standard stock solution, containing sorbitol-^13^C_6_ (approx. 7 µg) and heptadecane (approx. 9 µg) is added, using the multipurpose sampler. Then, silylation (37 °C/30 min) is performed just-in-time by addition of 40 µL silylation reagent. After silylation 200 µL of pyridine is added before the GC-injection by the Gerstel Multipurpose MPS2 sampler. A pooled sample produced by sampling an aliquot from all samples in the set was analysed a number of times with the samples in parallel to a number of blank samples. Each sample was only analysed once due to limited amount of sample material. All vials were analysed in random order. All data acquisition was performed on a system consisting of an Agilent 7890A gas chromatograph (Agilent Technologies, Santa Clara, CA, USA) with a Gerstel Multipurpose MPS2 autosampler (Gerstel GmbH & Co.KG, Mülheim an der Ruhr, Germany) interfaced to a LECO Pegasus time-of-flight mass spectrometer with an electron ionization (EI) source (LECO Corporation, St. Joseph, MI, USA). The GC was mounted with 30 m × 0.25 mmID × 0.25 µm 5%Phenyl-95%methyl-silicone capillary column, RTX5 (Restek, Bellefonte, PA, USA) with a 0.5 m similar precolumn. Injection was 1 µL with a split ratio of 1:20 in a split/splitless injector kept at 280 °C mounted with an Agilent Deactivated Split Taper Inlet Liner. The column was operated with a helium flow of constant ca. 1 mL/min, fine adjusted to maintain retention time for three internal standards within ± 0.5 s. The transfer line was maintained at 250 °C, and the oven temperature ramp initial 50 °C, followed by 10 °C/min to 320 °C, which is then kept for 10 min. The MS conditions were with −70 eV electron energy, ion source temperature of 250 °C, acquisition delay 180 s, acquisition rate 20 spectra/s and a mass range of *m/z* 70–1000. The data processing was performed using LECO ChromaTOF v.4.71.0.0 and GeneData Expressionist Refiner and Analyst version 10.5 (GeneData AG, Basel, Switzerland) LECO data files were loaded to GeneData Expressionist Refiner and individual isotopic masses were summed to nominal masses and subjected to a series of noise reduction including smoothing and signal intensity clipping steps. Peaks were detected and grouped and were assigned based on the AMDIS algorithm towards an in-house spectral library. Relative comparisons of profiles were based on responses calculated as a characteristic ion for the compound divided with a characteristic ion for the sorbitol-^13^C_6_ internal standard.

Multiblock toolbox version 0.2 for Matlab (http://www.models.life.ku.dk/MBToolbox) was used for the Multi Block Principal Component Analysis (MB PCA) model to integrate all metabolomics data across biofluids and tissues. Pareto-scaling and normalization to 1-norm were used on each block when modelling. 

PathVisio 3.2.4 was used to create metabolic pathway diagram in Figure 4 [33].

### 4.4. Metagenomics Data Generation and Analysis

DNA was extracted from fecal pellets using a PowerSoil^®^ DNA isolation kit (MoBio). Mechanical agitation was provided using a Mini-BeadBeater-1 (BiosSpec Products) for two one-minute pulses at the highest energy setting per sample. Amplicon sequencing libraries were generated targeting the bacterial 16srRNA gene V4 region using primers 515F and 806R with cycle parameters, 12 base Golay barcodes, and Illumina adaptor addition as detailed by the earth microbiome project 16S Illumina Amplicon Protocol. Libraries were sequenced on an Illumina MiSeq instrument 250 base in both directions through the 253 base V4 region. Sequences were processed for sequence error correction, chimera removal, and denovo OTU picking and taxonomic identification using the Mothur package following the MiSeq SOP [34]. Diversity was calculated in R (3.4.1, Vegan [35]) using the Shannon-index. The linear discriminant analysis effect size (LEfSe) [36] was used to identify differentially abundant taxa between HFD and LFD groups. Both Kruskal–Wallis and Wilcoxon rank-sum tests in LEfSe were used to identify significant differences. Taxa with a log-transformed LEfSe >2 and *p*-value < 0.05 was considered statistically significant. Significance values are reported in text, figures and figure legends. Principal Coordinates Analysis (PCoA) based on the Bray–Curtis distance was used to visualize the sample clusters and dissimilarity of microbial community due to diet and sampling time. PCoA was performed in R (3.4.1; Phyloseq [37]). 

## Figures and Tables

**Table 1:** Fold change of the metabolite levels in liver. A fold change of >1.5 or <0.66 was considered qualitatively important. Significance levels tested by one-way ANOVA with Benjamini–Hochberg correction. Abbreviations: LFD, low-fat diet; HFD, High-fat diet.

Metabolite	LFD	HFD
Acetate	1.00	1.10
Glucose	1.00	1.27
Pyruvate	1.00	1.14
Fumarate	1.00	2.95*
cis-Aconitate	1.00	0.83
Citrate	1.00	0.66
Malate	1.00	2.60 **
Succinate	1.00	1.65 *
Taurine	1.00	0.89
Oxaloacetate	1.00	1.78 *
Oxoglutarate	1.00	1.31

The false discovery rate-corrected *q* < 0.05 was considered significant: * *q* < 0.05, ** *q* < 0.01.

## Supplementary Materials

The following are available online at https://www.mdpi.com/2218-1989/10/3/80/s1, Figure S1. Multi-block PCA block scores and loadings, Table S1. Body and selected organ weights, Table S2. Fold change of the gene expression in liver. Significance levels tested by one-way ANOVA with Benjamini-Hochberg correction.
