# Supplementary material for: An Integrated Multi-Omics Analysis Defines Key Pathway Alterations in a Diet-Induced Obesity Mouse Model
Source: Metabolites. 2020 Feb 25;10(3):80. doi: 10.3390/metabo10030080 (PMC7143281; doi:10.3390/metabo10030080)
Supplement: Supplementary file 1 [file metabolites-10-00080-s001.pdf]

Supporting Table 1. Body and selected organ weights.

Mouse ID	Diet	Weight (g)			
		Body	Kidney	Liver	Spleen
1	LFD	32.4	0.383	1.11	0.064
2	LFD	29.5	0.381	1.05	0.05
3	LFD	30.1	0.316	1.04	0.057
4	LFD	33.7	0.379	1.24	0.067
5	LFD	28.3	0.314	0.93	0.044
6	LFD	30.2	0.32	1.06	0.058
7	LFD	32.7	0.357	1.20	0.055
8	LFD	31	0.325	1.03	0.062
9	LFD	36.4	0.374	1.29	0.065
10	LFD	31.4	0.35	1.05	0.059
11	HFD	49.2	0.4	1.81	0.07
12	HFD	35.9	0.337	0.86	0.055
13	HFD	50.8	0.441	2.27	0.084
14	HFD	51.4	0.405	1.93	0.058
15	HFD	49.5	0.473	1.15	0.103
16	HFD	48	0.474	1.32	0.098
17	HFD	49.1	0.367	1.82	0.075
18	HFD	50.1	0.493	1.47	0.062
19	HFD	47	0.406	1.89	0.072
20	HFDs	47	0.394	1.29	0.068

Supporting Table 2. Fold change of the gene expression in liver. Significance levels tested by one-way ANOVA with Benjamini-Hochberg correction. The false discovery rate-corrected *q* <0.05 was considered significant: * *q* < 0.05, ** *q* < 0.01

Metabolite	LFD	HFD
Mdh1	1	0.90
Mdh2	1	0.86*
Pcx	1	1.03
Pdha1	1	1.04
Pdha2	1	0.93
Pdhb	1	1.36*
Dlat	1	1.05
Dld	1	0.99
Pdhx	1	0.93
Pdk1	1	1.30
Pdk2	1	0.87
Pdk3	1	1.35
Pdk4	1	0.76
Cs	1	1.12
Aco2	1	0.88
Idh3a	1	1.10
Idh3b	1	0.88*
Idh3g	1	0.97
Idh2	1	0.87
Ogdh	1	1.36
Dlst	1	0.83*
Dld	1	0.99
Suclg1	1	0.78*
Suclg2	1	1.25*
Sdha	1	1.02
Sdhb	1	0.92
Sdhc	1	0.96
Sdhd	1	0.74*
Fh1	1	1.27*

Supporting figure 1

Multi-block PCA. Block scores (Blue: LFD; Green: HFD) and loadings; A-B) metadata, C-D) NMR urine metabolomics, E-F) NMR serum metabolomics, G-H) HRMAS NMR liver metabolomics on intact tissue, I-J) NMR metabolomics of liver methanol:water fraction, K-L) NMR metabolomics of liver $CHCl_3$ fraction, M-N) NMR metabolomics of adipose methanol:water fraction, O-P) NMR metabolomics of adipose $CHCl_3$ fraction, Q-R) LC-MS metabolomics of urine, S-T) LC-MS metabolomics of serum, U-V) LC-MS metabolomics of liver methanol:water fraction, W-X) LC-MS metabolomics of muscle methanol:water fraction, Y-Z) GC-MS metabolomics of liver methanol:water fraction, AA-BB) GC-MS metabolomics of muscle methanol:water fraction. Assignments: 1, Body weight; 2, Mass of liver; 3, Blood total bilirubin; 4, Blood calcium; 5, Blood inorganic phosphorous; 6, Blood total Na; 7, Blood total Cl; 8, Trimethylamine; 9, Citrate; 10, Succinate; 11, Alanine; 12, Lipids; 13, Lactate; 14, Malate; 15, Taurine; 16, Choline; 17, Leucine; 18, 3-hydroxybutyrate; 19, Acetate; 20, Glycerol; 21, Glutaconic acid*; 22, Vinylacetylglycine*; 23, Phenylalanyltryptophan*; 24, N-Heptanoylglycine*; 25, Fumaric acid*; 26, cis-Aconitic acid*; 27, Indoleacrylic acid*; 28, Imidazole acetol-phosphate*; 29, PC(14:0/20:2)*; 30, PC(18:1/22:6)*; 31, Phosphate; 32, Threonine; 33, Glycine; 34, Phenylalanine; 35, Isoleucine; 36, Creatinine; 37, 2-oxo-proline. *: tentative assignments based on molecular weight.

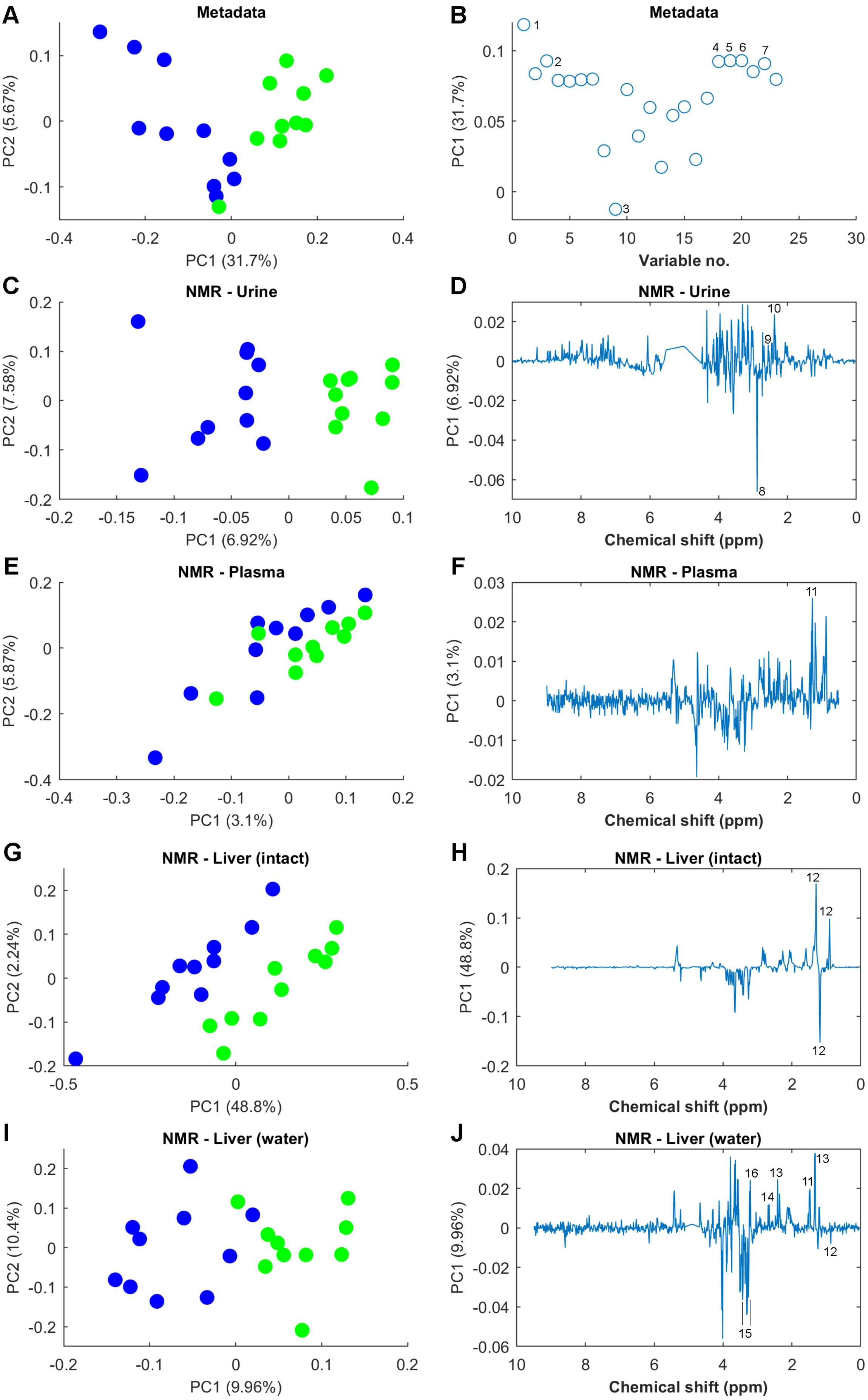
A
Metadata
PC2 (5.67%)
PC1 (31.7%)
B
Metadata
PC1 (31.7%)
Variable no.
1
2
3
4 5 6
7
C
NMR - Urine
PC2 (7.58%)
PC1 (6.92%)
D
NMR - Urine
PC1 (6.92%)
Chemical shift (ppm)
10
9
8
E
NMR - Plasma
PC2 (5.87%)
PC1 (3.1%)
F
NMR - Plasma
PC1 (3.1%)
Chemical shift (ppm)
11
G
NMR - Liver (intact)
PC2 (2.24%)
PC1 (48.8%)
H
NMR - Liver (intact)
PC1 (48.8%)
Chemical shift (ppm)
12
12
12
I
NMR - Liver (water)
PC2 (10.4%)
PC1 (9.96%)
J
NMR - Liver (water)
PC1 (9.96%)
Chemical shift (ppm)
16
13
14
11
13
12
15

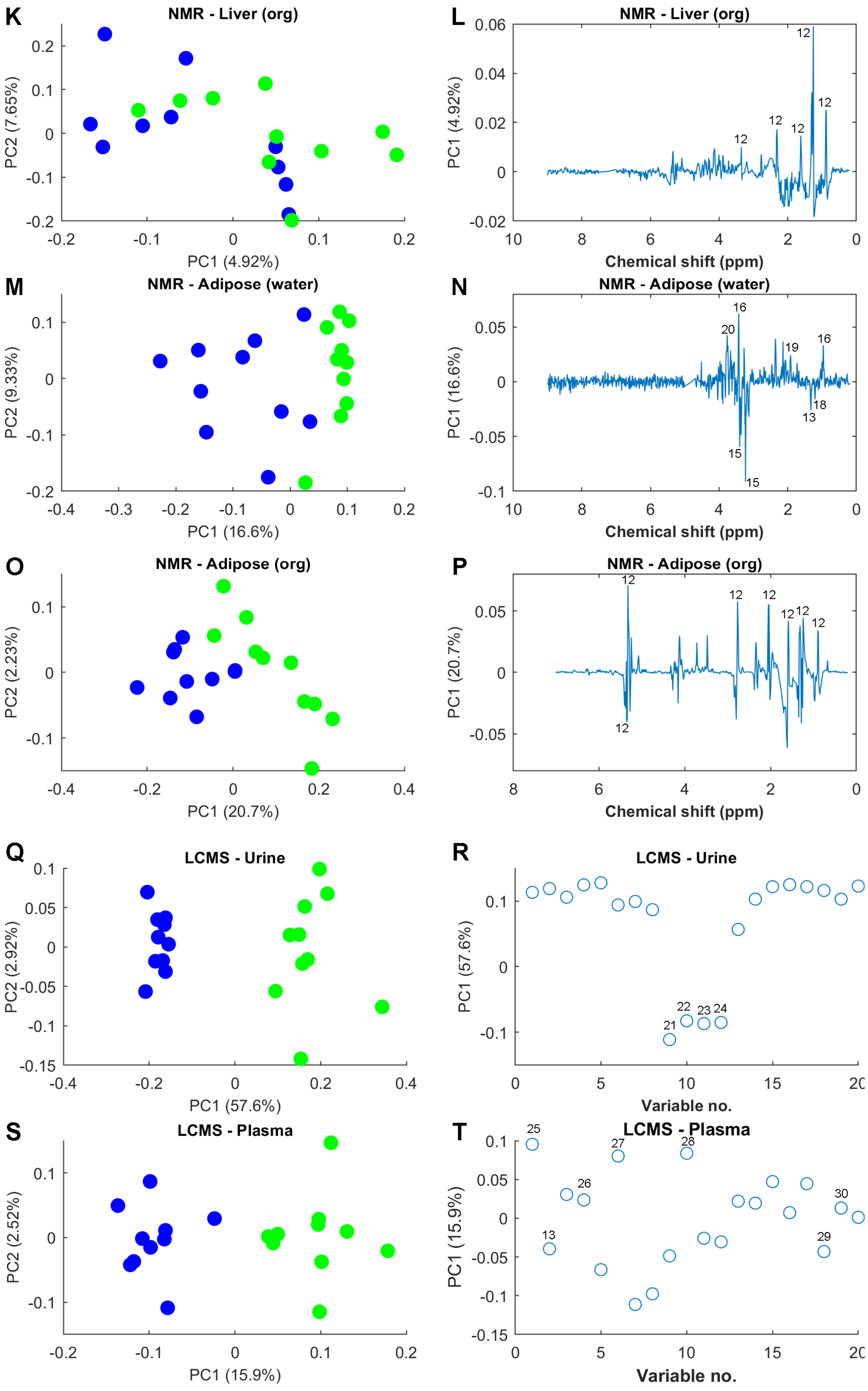
K
NMR - Liver (org)
PC2 (7.65%)
PC1 (4.92%)
L
NMR - Liver (org)
PC1 (4.92%)
Chemical shift (ppm)
M
NMR - Adipose (water)
PC2 (9.33%)
PC1 (16.6%)
N
NMR - Adipose (water)
PC1 (16.6%)
Chemical shift (ppm)
O
NMR - Adipose (org)
PC2 (2.23%)
PC1 (20.7%)
P
NMR - Adipose (org)
PC1 (20.7%)
Chemical shift (ppm)
Q
LCMS - Urine
PC2 (2.92%)
PC1 (57.6%)
R
LCMS - Urine
PC1 (57.6%)
Variable no.
S
LCMS - Plasma
PC2 (2.52%)
PC1 (15.9%)
T
LCMS - Plasma
PC1 (15.9%)
Variable no.

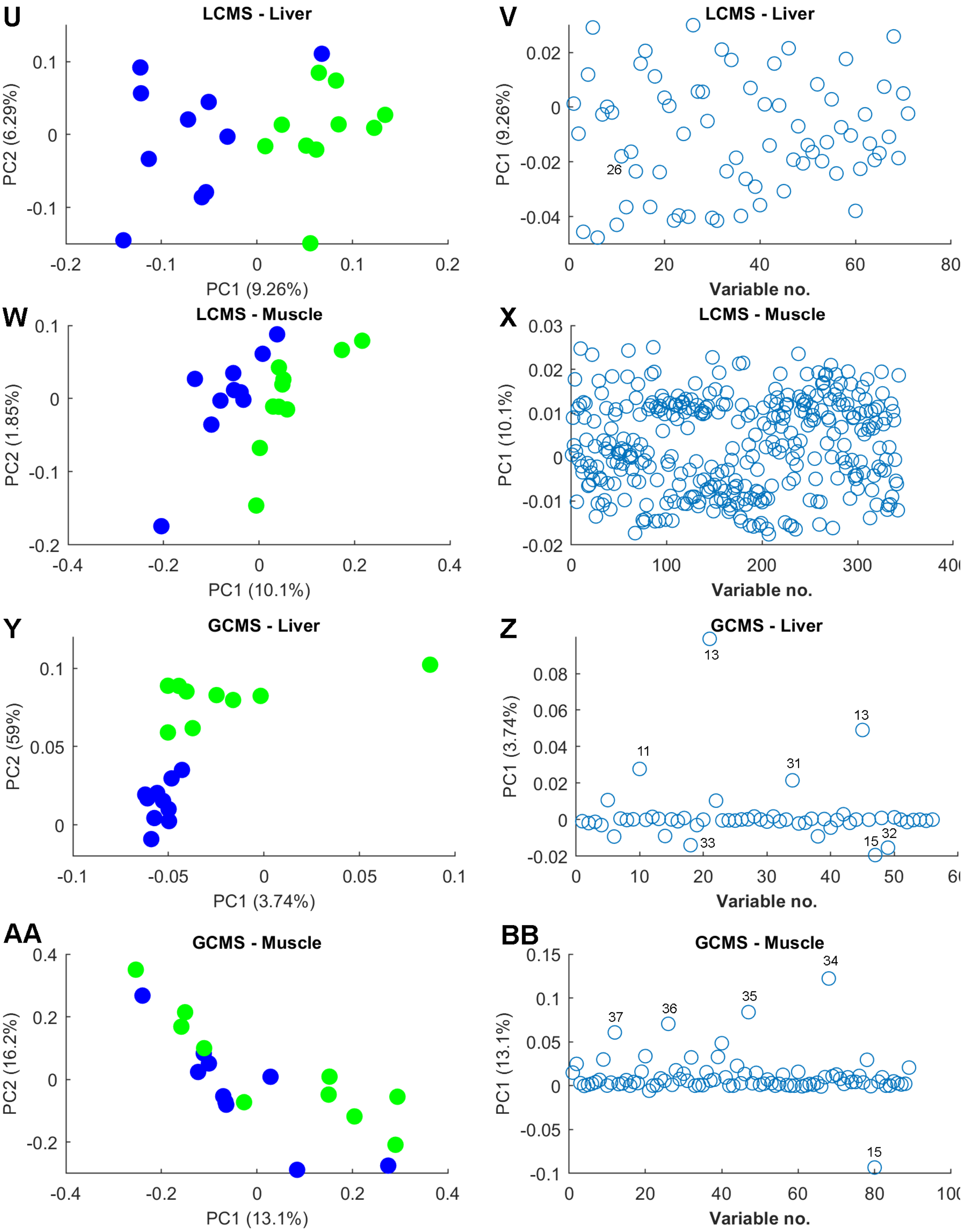

U
LCMS - Liver
PC2 (6.29%)
PC1 (9.26%)
0.1
0
-0.1
-0.2
-0.1
0
0.1
0.2
V
LCMS - Liver
PC1 (9.26%)
Variable no.
0.02
0
-0.02
-0.04
26
0
20
40
60
80
W
LCMS - Muscle
PC2 (1.85%)
PC1 (10.1%)
0.1
0
-0.1
-0.2
-0.4
-0.2
0
0.2
0.4
X
LCMS - Muscle
PC1 (10.1%)
Variable no.
0.03
0.02
0.01
0
-0.01
-0.02
0
100
200
300
Y
GCMS - Liver
PC2 (59%)
PC1 (3.74%)
0.1
0.05
0
-0.1
-0.05
0
0.05
0.1
Z
GCMS - Liver
PC1 (3.74%)
Variable no.
0.08
0.06
0.04
0.02
0
-0.02
13
11
31
13
33
15
32
0
10
20
30
40
50
AA
GCMS - Muscle
PC2 (16.2%)
PC1 (13.1%)
0.4
0.2
0
-0.2
-0.4
-0.2
0
0.2
0.4
BB
GCMS - Muscle
PC1 (13.1%)
Variable no.
0.15
0.1
0.05
0
-0.05
-0.1
34
37
36
35
15
0
20
40
60
80
